# Effects of Dexmedetomidine on Diaphragm Activity Measured by Ultrasonography in Spontaneously Breathing Patients

**DOI:** 10.7150/ijms.76495

**Published:** 2022-09-21

**Authors:** Hye Joo Yun, Dakyung Hong, Sung Jun Kim, Hye Won Chung, Hyun Sik Chung

**Affiliations:** Department of Anesthesiology and Pain Medicine, Eunpyeong St. Mary's Hospital, College of Medicine, The Catholic University of Korea, Seoul, Republic of Korea

**Keywords:** Dexmedetomidine, Deep sedation, Diaphragm, Respiratory complication, Respiratory muscles, Ultrasonography.

## Abstract

**Background:** Diaphragm movement is well correlated with inspired volume of the lung. Dexmedetomidine (DEX) has less effect on respiratory functions than other sedatives. The objective of this study was to investigate diaphragmatic movement using ultrasound (US) during DEX infusion for sedation in spontaneously breathing patients undergoing unilateral upper limb surgery.

**Methods:** A total of 33 consecutive patients were enrolled in this study. Patients were sedated using DEX with ipsilateral axillary brachial nerve plexus block. Diaphragmatic activity was evaluated using diaphragmatic thickening at end-inspiration (TEI), diaphragmatic thickening at end-expiration (TEE), and diaphragmatic thickening fraction (DTF) measured by diaphragmatic US at three time-points; T0, baseline; T1, after DEX sedation; and T2, after DEX recovery. Supplementary oxygen was applied with a simple mask at 5 L/min. Peripheral oxygen saturation (SpO_2_), end tidal CO_2_ (EtCO_2_), and respiratory rate (RR) were recorded.

**Results:** TEI and TEE showed no significant changes during the study period (*P* = 0.394 and *P* = 0.205, respectively). DTF was maintained at both T0 and T1 (*P* = 1.000). At recovery after DEX infusion discontinued, DTF was increased by 3.85%, although such increase was not statistically significant (T0 vs. T2, *P* = 0.525). SpO_2_ remained above 99% and EtCO_2_ remained below 36 mmHg. Desaturation episodes were not observed during the study period.

**Conclusions:** Results of this study showed that DEX sedation did not affect the diaphragmatic movement in situation of decreased RR induced by DEX. This finding implies that DEX-induced sedation does not result in clinically significant respiratory depression.

## Introduction

The diaphragm plays an important role in maintaining appropriate respiration in humans. It is the dominant muscle for respiration 1. It is known that diaphragmatic movement is well-correlated with inspired volume of lung in both supine and sitting positions 2. Thus, assessment of diaphragmatic movement could be useful for measuring respiratory functions of patients.

Several tools for assessing diaphragmatic movement are available, including radiology and electromyography. Transitional plain radiography is relatively insensitive due to wide normal variability of diaphragm position. Fluoroscopy also has limitation to quantitate, including paradoxical movement on sniffing. In addition, fluoroscopy has a risk to exposures to significant radiation like computed tomography. Transportation is also needed for taking the patient to the room equipped for applying radiology. Magnetic resonance imaging also needs to transport the patient to the room equipped. It also has a high cost. Electromyography as a non-imaging test is technically challenging to perform including the risk of pneumothorax 3. Magnetic phrenic stimulation is considered as an evoked maneuver. It has an advantage as a reference method. However, it is only available in expert centers that can afford to use recent techniques 4, 5. On the other hand, neuromuscular ultrasound (US) is a simple, portable, non-invasive technique without any radiation risk to evaluate diaphragmatic structure and activity. It was first introduced by Cohen *et al*. 6 in 1969. It has advanced recently to allow high resolution images. US has been shown to have an accuracy similar to most other imaging modalities for assessing diaphragmatic activity 3, 7, 8.

Sedation for procedures is a rising demand both inside and outside the operating room. This in turn has led to concern over potential life-threatening complications such as respiratory depression caused by sedatives 9. Dexmedetomidine (DEX) as an α_2_-adeonoceptor agonist is an alternative drug of choice for sedation to achieve effective sedation while avoiding respiratory complications. In addition, DEX has stable hemodynamics by vagomimetic actions of peripheral postsynaptic α_2_b-receptors in the vascular smooth cells of resistant vessels 10. However, studies evaluating the effect of DEX on the diaphragmatic activity especially using US as a non-invasive assessment method are lacking, although effects of some other sedatives on diaphragmatic activity have been evaluated 11-13.

In this respect, the aim of this observational prospective study was to investigate the effect of DEX on respiratory muscles, especially hemi-diaphragmatic activity, by US in spontaneously breathing patients under deep DEX sedation undergoing elective upper limb surgery with ipsilateral axillary brachial plexus nerve block.

## Methods

This study was approved by the Institutional Review Board (IRB) of our institution (PC19OISI0134). All enrolled patients provided written informed consent. A total of 34 consecutive patients between May 2020 and October 2021 were enrolled in this study. All patients underwent unilateral elective limb surgery with ipsilateral axillary brachial nerve plexus block under deep dexmedetomidine sedation. Inclusive criteria were American Society of Anesthesiologists physical status classification (ASA) grades I and II, age ≥ 19 yrs, and normal cardiac and pulmonary function. Exclusion criteria were: neurologic or muscular diseases including peripheral neuropathy and central nervous system disorders, receiving anticholinergic drugs, diaphragm palsy on chest X-rays, and lack of informed consent.

The block was performed at ipsilateral axillary brachial nerve plexus using US. The patient's limb of ipsilateral side for surgery was abducted to 90 degrees, with the elbow flexed to 90 degrees. An 8 to 14 MHz linear transducer was placed in a transverse plane at the lateral border of the pectoralis major muscle and the pulsating axillary artery was visualized. A 22-gauge needle (UniPlex^®^NanoLine^®^, PAJUNK^®^ Holding GmBH, Geisingen, Germany) was used to administer local anesthetic to axillary nerves including radial, median, ulnar, and mulsculocutaneous nerves surrounding the axillary artery. For local anesthetic, a total 35 ml of 0.5 % ropivacaine hydrochloride (Naropin 0.75%, Mitsubishi Tanabe Pharma Co., Osaka, Japan) was used to perform axillary brachial plexus block. An axillary brachial plexus block was performed on the patient's left side for ipsilateral upper limb surgery.

Sedation was provided using intravenously continuous infusion of DEX (dexmedetomidine hydrochloride 200mcg/50ml, Precedex premix injection, Pfizer, NY, USA) along with a loading dose by anesthesiologist under guidance of the Oberver's Assessment of Alertness/Sedation (OAA/S) scale and bispectral index (BIS) 14. DEX was administered at 1mcg/kg for 10 mins as a loading dose, followed by continuous infusion of DEX at a dose of 0.3 to 0.8 mcg/kg/hr to maintain level 1 according to the OAA/S scale.

Supplementary oxygen was applied at a rate of 5 L/min via a simple mask. Peripheral oxygen saturation (SpO_2_), end tidal CO_2_ (EtCO_2_), and respiratory rate (RR) were monitored. All hypoventilation episodes were recorded. Ultrasonographic hemi-diaphragmatic measurement was performed by a single anesthesiologist who was well trained in using LOGIQ Ultrasound (LOGIQ E10, GE medical systems ultrasound and Primary care diagnostic, LLC., Wauwatosa, WI, USA). All diaphragmatic measurements were obtained using millimeters as a unit in a supine position on the patient's right side which was the contralateral side of axillary block and surgery.

Diaphragmatic thickening was measured using M-mode in the zone of apposition (ZOA) to the rib cage from 8^th^ to 9^th^ intercostal space (Figure 2). The US transducer was placed at the anterior axillary line between the 8^th^ and 9^th^ ribs. At that time, real-time diaphragmatic movement could be recorded in M-mode. The diaphragmatic muscle was observed as an-echogenic central layers between two echogenic layers consisting of the diaphragmatic pleura and peritoneum using linear 10 MHz probe. Diaphragmatic thickening was measured by placing electronic calipers inside the two hyper-echoic lines. Two different phases of diaphragmatic thickening were measured at end-inspiration (TEI), and end-expiration (TEE). Diaphragmatic thickening faction (DTF) representing diaphragmatic efficiency was calculated as follows:

DTF = [(TEI - TEE) / TEE]

Diaphragmatic measurements were performed at three time-points: T0, before axillary brachial nerve plexus block and DEX administration; T1, 1 min after reaching level 1 of OAA/S scale by DEX administration following axillary brachial nerve plexus block; and T2, 5 mins after reaching level 5 of OAA/S scale after discontinuing DEX. All measurements were obtained on the right side of ZOA with breathing spontaneously.

The primary endpoint was the change in diaphragmatic activity from sedation with DEX in patients with spontaneously breathing. Secondary endpoints were changes in respiration related parameters including incidence of hypoventilation episodes.

Based on preliminary results in a pilot study, a sample size of 34 patients would have 80 % power to detect to minimum 0.08 mm of TEE difference and a maximum SD difference of 0.15 considering an alpha error < 0.05 and a dropout rate of 10%. Shapiro-Wilk's test was performed to assess normal data distribution. Time-sequential change according to the three time points was subjected to repeated measures analysis of variance (ANOVA). Post-hoc test was performed by Bonferroni correction. Data are expressed as mean ± standard deviation (SD) or numbers (percentage) as appropriate. All tests were two-sided, and *P* < .05 was deemed statistically significant. All statistical analyses were performed using R version 4.1.2 (R Foundation for Statistical Computing, Vienna, Austria).

## Results

**Figure 1:**
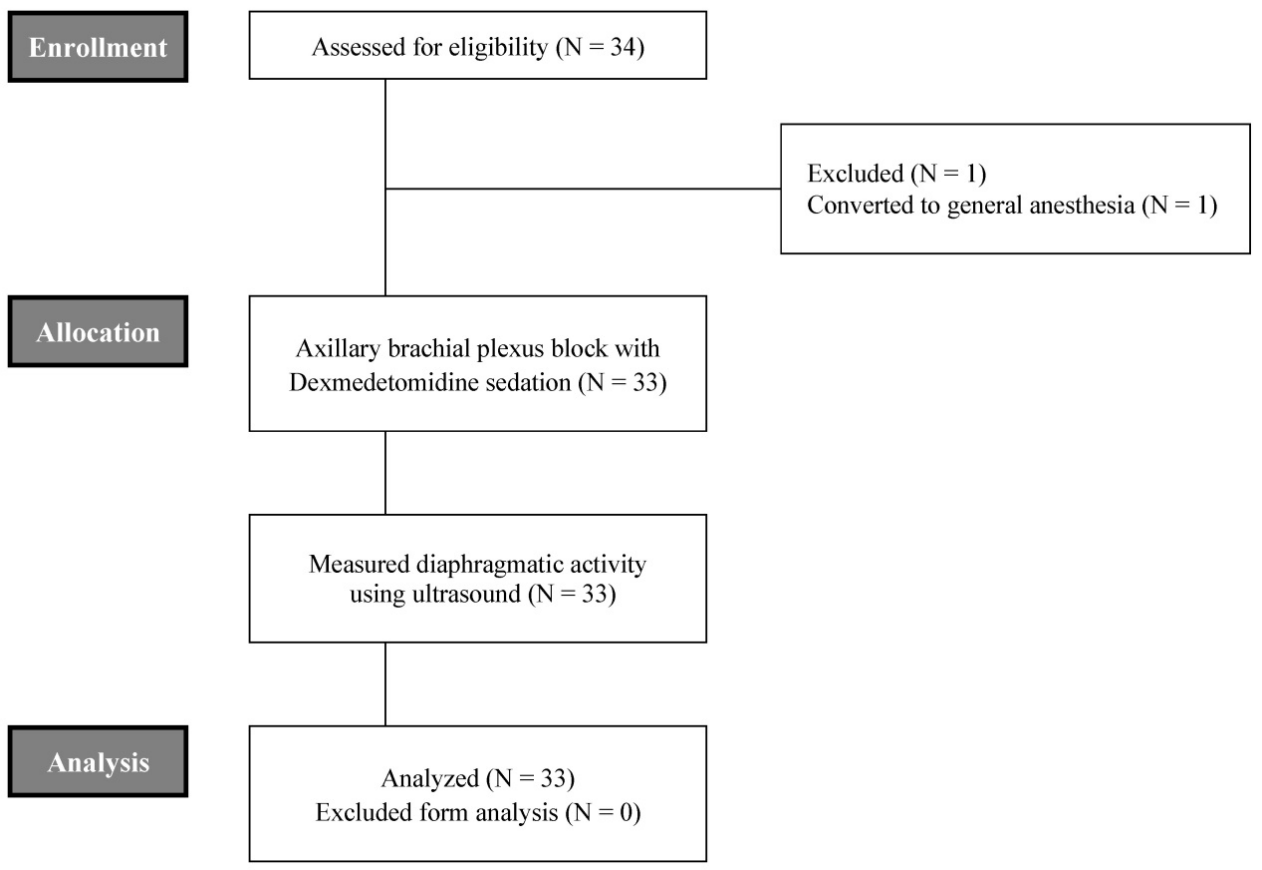
CONSORT flowchart of this study.

**Figure 2:**
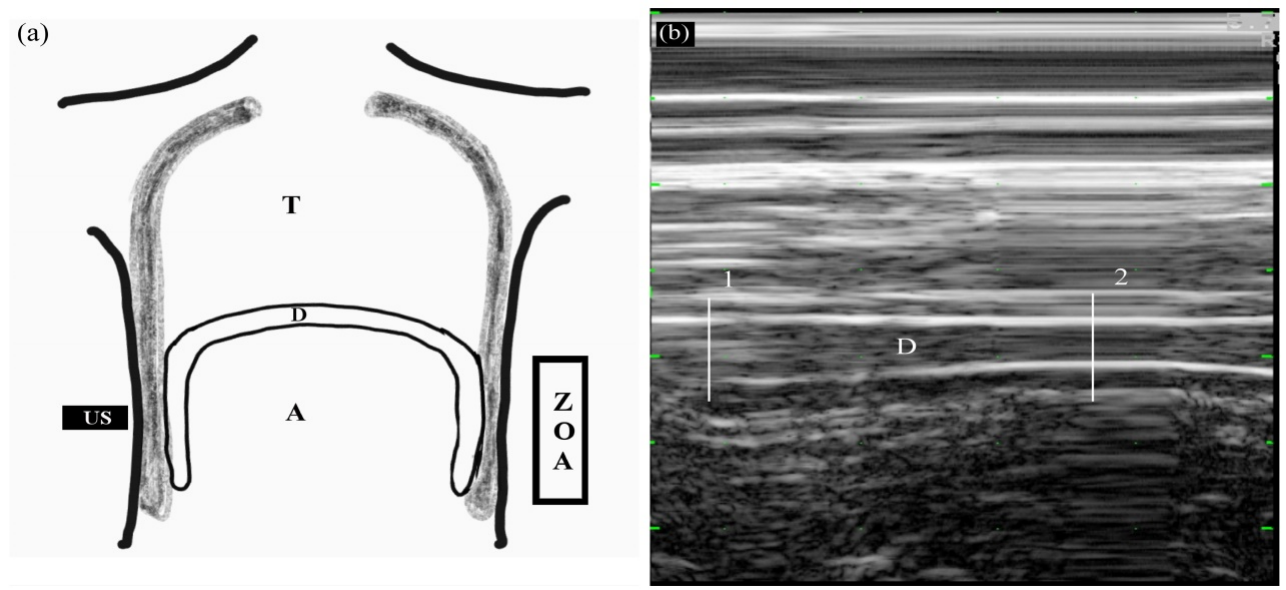
Measuring diaphragmatic thickening using M-mode in the zone of apposition (ZOA) to the rib cage from 8^th^ to 9^th^ intercostal space. (a) Frontal section of the chest wall illustrating the functional anatomy of the diaphragm. A, abdomen; D, diaphragm; T, thoracic cage; US, ultrasound probe; ZOA, zone of apposition. (b) Recording of changes in diaphragm thickening during spontaneous breathing using M-mode tracing. 1, end-inspiration; 2, end-expiration; D, diaphragm.

A total of 34 patients were enrolled for the present study, excluding 1 case due to conversion to general anesthesia as consequence of incomplete axillary brachial plexus block (Figure 1). The mean age of these patients was 39 ± 14 years. Males were predominant (57.6 %). The mean body mass index (BMI) of all patients was 24.4 ± 3.8. ASA grades I and II accounted for 78.8 % and 21.2 %, respectively. With respect to underlying diseases, three patients had hypertension and one patient had diabetes mellitus. Mean total operation time and recovery time for level 5 from level 1 of OAA/S scale after DEX infusion was stopped were 48.1 ± 25.4 mins and 82.9 ± 13.3 mins, respectively (Table 1).

**Figure 3:**
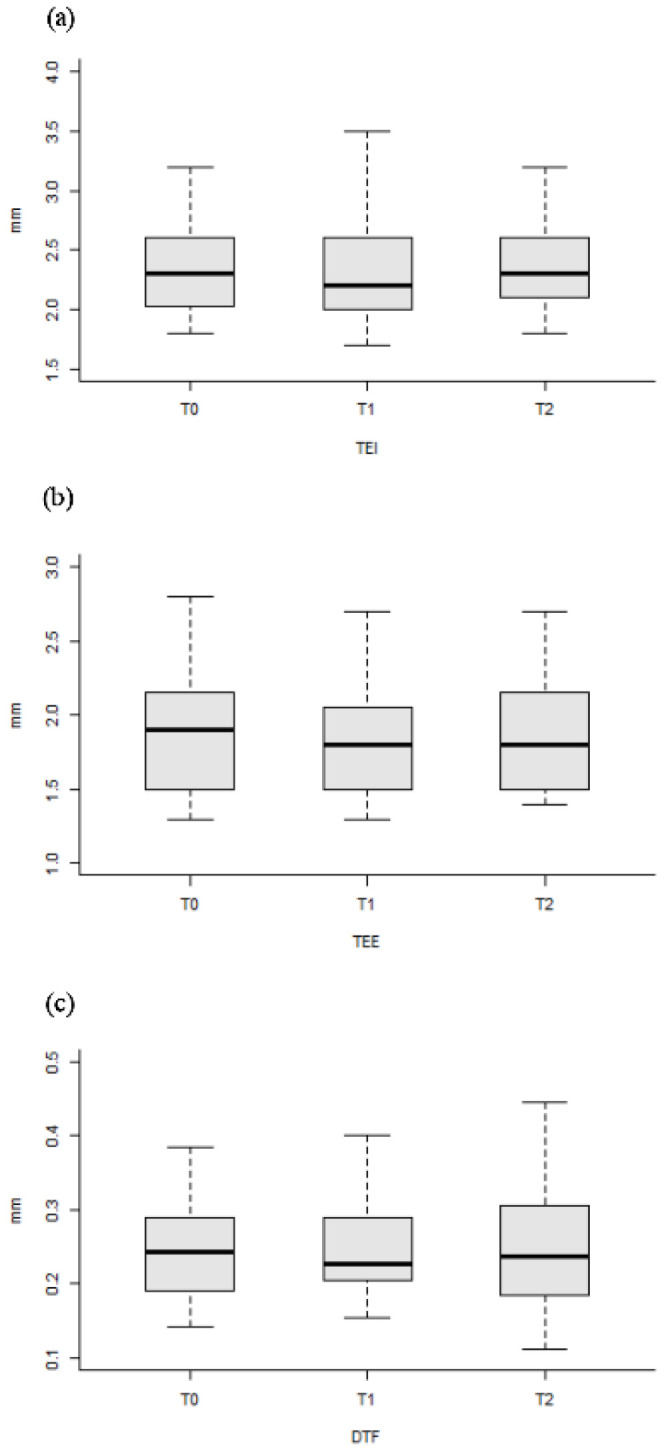
Changes in diaphragmatic activity according to the three time points. Medians (horizontal bar), 25^th^ and 75^th^ percentiles, and maximum and minimum (whiskers) of selected features are shown. T0, before axillary block and dexmedetomidine infusion; T1, 1 min after reaching level 1 on the OAA/S scale after ipsilateral axillary block and dexmedetomidine infusion; T2, 5 min after reaching level 5 on the OAA/S scale in postanesthetic care unit. TEI, diaphragmatic thickening at end-inspiration; TEE, diaphragmatic thickening at end-expiration; DTF, diaphragmatic thickening fraction.

Changes in all variables including diaphragmatic activity according to DEX administration are shown in Table 2 and Figure 3. TEI and TEE showed no significant differences throughout the three time points. DTF showed no significant differences among the three time points either. TEI and TEE showed no significant changes during DEX infusion, although values of TEI and TEE were slightly reduced (T0 vs. T1, reduced by 1.68 % and 20.9 %, *P* = 0.137 and *P* = 0.119, respectively). DTF values showed no significant (*P* = 0.774) change during the study period either, although its value was slightly increased by 3.85 % after recovery from sedation with DEX (T0 vs. T2, *P* = 0.525).

The level of mean SpO_2_ was consistently above 99 %. EtCO_2_ was always below 36 mmHg. No desaturation episodes were developed in any patients during the study period. RR was significantly decreased by 23.4 % during DEX infusion (T0 vs. T1, *P* < 0.05). Decreased RR was maintained after recovery from DEX sedation (T0 vs. T2,* P* < 0.01). HR was also significantly decreased by 19.7 % at T1 (T0 vs. T1, *P* < 0.01). The decrease of HR was maintained at T2 (T0 vs. T2, *P* < 0.01). Mean blood pressure maintained at T1 was significantly decreased by 16.8 % at T2 (T0 vs T2, *P* < 0.01). Mean BIS was significantly decreased by 36.2 % at T1 (T0 vs. T1, *P* < 0.01). It recovered to be above 90 pts at T2 after DEX infusion was discontinued (T1 vs. T2,* P* < 0.01).

## Discussion

The main finding of this study was that the diaphragmatic movement was not significantly changed by DEX infusion to reach deep sedation (level 1 of OAA/S scale). The RR was significantly decreased at time-point during DEX infusion (level 1 of OAA/S scale). It did not return to the value of baseline after DEX sedation was recovered (level 5 of OAA/S scale) after DEX infusion was stopped. This suggests that DEX infusion can maintain diaphragmmatic movement regardless of RR depression induced by DEX.

DEX is a selective and potent α_2_-adrenoreceiptor agonist that has anxiolytic, sedating, and analgesic effects 15. An outstanding feature of DEX-based sedation is that it has minimal influences on patient's respiration functions. This property of DEX regarding respiration functions makes DEX an interesting alternative sedative in many procedures including sedation for regional anesthesia. Side effects for DEX are mainly restricted to hemodynamic changes, including hypertension, bradycardia, and hypotension owing to pre- and post-synaptic α_2_-receptor activation known to cause vasoconstriction, vasodilatation, and reflex bradycardia 16, 17.

Regarding respiratory effects of DEX, it has little respiratory depression as the consensus under debated previous study 15. Results of this study confirmed that DEX did not cause respiratory depression based on diaphragmatic activity measured by US, although RR was decreased after infusion of DEX. The decrease of RR was suspended after recovery from DEX sedation. In terms of DTF, it was slightly increased at T2 then that at both T0 and T1. However, such increase was not statistically significant. It was assumed the patients could enforce their respiration after awakening from DEX sedation. The EtCO_2_ was below 36 mmHg during the study period. The value of EtCO_2_ is lower than normal range. This might be due to the use of open oxygen delivery system for oxygen supply with a simple mask. Therefore, trends of EtCO_2_ are more valuable than absolute values of EtCO_2_ in this study. Trends of EtCO_2_ were maintained in enrolled patients during the study period, indicating that their respiratory functions were maintained during the study period. Moreover, the SpO_2_ maintained above 99 % during the study period. Thus, DEX is a safe sedative in both inside and outside the operating room in terms of respiration depression which is the most prominent adverse effect of any sedatives.

With respect to parameters related to diaphragmatic movement measured by US, we used the calculated DTF to compare DEX-induced effect on the diaphragm. DTF is known to be correlated with pressure generating capacity of the diaphragm even in spontaneous breathing 18, although the correlation of DTF with lung volume has been debated 19-21. Additionally, we measured diaphragmatic thickness using ZOA. Measurements of diaphragmmatic thickness using ZOA are reliable and reproducible with intra-observer or inter-observer reproducibility 20, 22, 23. We did not measure diaphragmatic excursions, one of widely used parameters related to diaphragm. It would be advantageous to measure diaphragmatic movement in spontaneous breathing patients 24. These parameters measured by US are acquired using one-dimensional images, whereas diaphragmatic movement is three-dimensional displacement of diaphragmatic muscles. Thus, differences in dimensions between US images and muscle contraction *in vivo* should be considered 25.

This study has some limitations. First, we did not use conventional methods to investigate diaphragmatic function, including transdiaphragmatic pressure (Pdi) measurement, phrenic nerve stimulation, or fluoroscopy 26. These methods are uncomfortable and cumbersome due to their invasiveness. In addition, they require specific technique and equipment 4. Second, we did not investigate inter-observer reproducibility of diaphragmatic US in measuring thickness as several other studies have already studied this parameter 19, 27. In this study, placement of the patients in the supine studied using the right diaphragm because some studies have shown that the right hemi-diaphragm is the easiest one to assess this muscle 20, 28. However, a supine position would be a less inherent resolution for ultrasound than a semi-recumbent position which is a usual position used for diaphragmatic ultrasound. A semi-recumbent position could obtain better diaphragm thickness by reducing abdominal pressure to diaphragm than a supine position 29. To overcome these limitations, a single well-trained anesthesiologist assessed the right side of the hemidiaphragm to improve accuracy and minimize an operator-dependent bias. Third, we only studied ASA I and II patients with normal respiratory functions. Further studies are needed to evaluate effects of these complex physiological changes on patients with pre-existing respiratory complications. Finally, the potential effect of airway obstruction on diaphragmatic motion has been raised as a concern 30. Although dexmedetomidine may cause airway obstruction during deep sedation, we did not observe snoring in any of our patients during the procedure. In addition, spontaneous breathing was always maintained. De-saturation episodes were not observed in this study. Mean values of EtCO_2_ were always below 36 mmHg. Such trends were maintained during DEX infusion 31.

In conclusion, this study confirmed that the diaphragmatic movement assessed using US was maintained during sedation with DEX regardless of RR change. This finding implies that DEX-induced sedation does not result in clinically significant respiratory depression, although DEX affects respiratory functions by decreasing RR and minute ventilation. Thus, DEX is a safe and useful drug for sedation without causing significant respiratory depression as evidenced by diaphragmatic US.

## Figures and Tables

**Table 1:** Demographic data of the study population.

Variables		N = 33		
Age (years)		39	±	14
Gender: male, N (%)		19		(57.6)
BMI (kg/m^2^)		24.4	±	3.8
ASA: I/II		26 (78.8)	/	7 (21.2)
Hypertension, N (%)		3		(9.1)
DM, N (%)		1		(3.0)
Operation time (mins)		48.1	±	25.4
Recovery time (mins)^a^		82.9	±	13.3

Data are presented as mean ± standard deviation or numbers (proportion). ASA, American society of anesthesiologists physical status classification; BMI, body mass index; DM, diabetes mellitus ^a^Time to level 5 form level 1 of OAA/S scale after dexmedetomidine infusion was stopped.

**Table 2:** Changes in variables according to the three time points.

Variables		T0				T1				T2				*P* Value^a^
TEI (mm)		2.38	±	0.42		2.34	±	0.45		2.37	±	0.38		0.394
TEE (mm)		1.91	±	0.43		1.87	±	0.39		1.90	±	0.42		0.205
DTF		0.260	±	0.093		0.260	±	0.082		0.272	±	0.135		0.774
SpO_2_ (%)		99.5	±	1.3		99.7	±	1.0		99.1	±	1.0		0.114
EtCO_2_ (mmHg)		32.2	±	3.5		32.1	±	3.9		31.8	±	3.8		0.905
RR (bpm)		19.7	±	4.3		18.0	±	3.9		15.1	±	3.3^*†^		<0.001
HR (bpm)		66	±	10		53	±	8^*^		54	±	8^*^		<0.001
MBP (mmHg)		95	±	11		93	±	12		79	±	11^*†^		<0.001
BIS (pts)		94	±	3		60	±	15^*^		91	±	6^*†^		<0.001

Data are presented as mean ± standard deviation. T0, before axillary block and dexmedetomidine infusion; T1, 1 min after reaching level 1 on the OAA/S scale after ipsilateral axillary block and dexmedetomidine infusion; T2, 5 min after reaching level 5 on the OAA/S scale in postanesthetic care unit. TEI, diaphragmatic thickening at end-inspiration; TEE, diaphragmatic at thickening end-expiration; DTF, diaphragmatic thickening fraction; SpO2, peripheral oxygen saturation; EtCO2, end-tidal CO2; RR, respiratory rate; HR, heart rate; MBP, mean blood pressure; BIS, bispectral index. ^a^*P* values were results from repeated measures ANOVA. ^*^P value < 0.05 vs. T0 and ^†^*P* value < 0.05 vs. T1 with post-*hoc* Bonferroni test.

## Abbreviations

DEX: dexmedetomidine
EtCO_2_: end tidal CO_2_
DTF: diaphragmatic thickening fraction
TEE: diaphragmatic thickening at end-expiration
TEI: diaphragmatic thickening at end-inspiration
OAA/S: Observer's Assessment of Alertness/Sedation
US: ultrasound
RR: respiratory rate
ZOA: zone of apposition
